# Bilirubin Oxidation Products and Cerebral Vasoconstriction

**DOI:** 10.3389/fphar.2018.00303

**Published:** 2018-04-27

**Authors:** Robert M. Rapoport

**Affiliations:** Department of Pharmacology and Systems Physiology, College of Medicine, University of Cincinnati, Cincinnati, OH, United States

**Keywords:** bilirubin oxidation products, BOXes, cerebral vasoconstriction, subarachnoid hemorrhage, endogenous BOXes, exogenous BOXes

## Abstract

Key evidence in support of the hypothesis that bilirubin oxidation products (BOXes) contribute to the vasoconstriction associated with subarachnoid hemorrhage (SAH) are the (1) presence of BOXes in cerebral spinal fluid from SAH patients and (2) ability of one or more BOXes to elicit vasoconstriction. We critically evaluate this key evidence, detail where gaps remain, and describe recent approaches that will address these gaps.

## Introduction

Efficacious treatment remains elusive for mitigating the devastating neurologic deficit associated with subarachnoid hemorrhage (SAH; Grasso et al., 2017). The complexity of identifying potential treatments is exasperated by the multiple impactful factors which negatively impact neurologic outcome (Foreman, 2016; Geraghty and Testai, 2017).

To understand the relative roles of these multiple factors in SAH-induced pathobiology the involvement of these factors has been differentiated by the time courses for their appearances relative to the initiation of SAH: acute, early, and late (Geraghty and Testai, 2017). Amongst the late developing contributory factors thought to negatively impact neurologic outcome is spasm of cerebral arteries (Crowley et al., 2008; Siasios et al., 2013; Foreman, 2016; Flynn et al., 2017; Geraghty and Testai, 2017).

**FIGURE 1:**
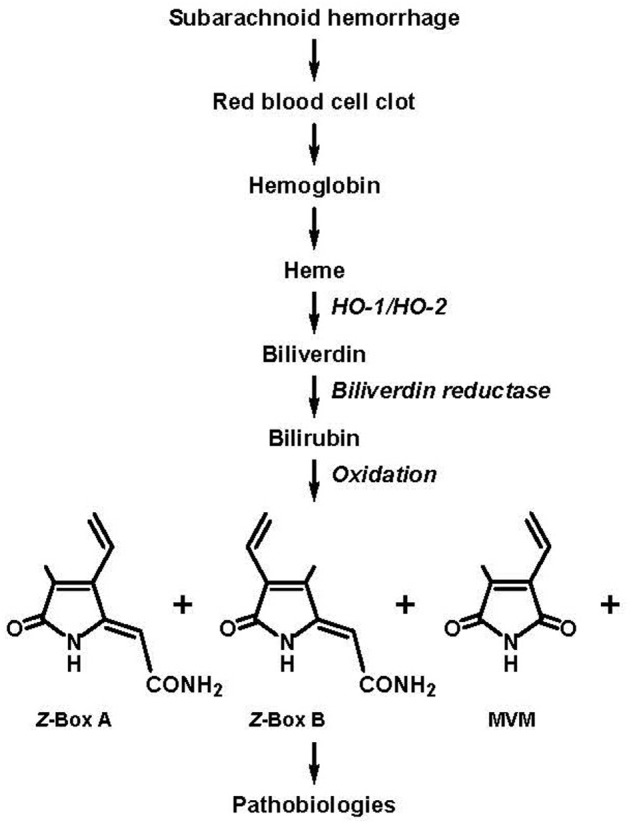
Subarachnoid hemorrhage (SAH) results in the accumulation of blood in the cerebral spinal fluid (CSF) and the eventual formation of bilirubin oxidation products (BOXes) through the following series of events: lysis of red blood cells in the blood clot, release of heme, oxidation of heme by heme oxygenase 1 (HO-1) and heme oxygenase 2 (HO-2) to form biliverdin, reduction of biliverdin by biliverdin reductase to form bilirubin, oxidation of bilirubin (and oxidation of heme and biliverdin) by oxygen free radicals to form BOXes. BOXes, which includes *Z*-BOX A (4-methyl-5-oxo-3-vinyl-(1,5-dihydropyrrol-2-ylidene)acetamide), *Z*-BOX B (3-methyl-5-oxo-4-vinyl-(1,5-dihydropyrrol-2-ylidene)acetamide), MVM (4-methyl-3-vinylmaleimide) and other compounds cause cerebral pathobiologic effects including spasm of the vasculature, increased stress in neural and vascular tissue, and cellular damage (Clark et al., 2004; Clark and Sharp, 2006; Pyne-Geithman et al., 2013; figure adapted from Clark and Sharp, 2006; specification of the *Z* regio-isomers of BOX A and BOX B based on Seidel et al., 2015).

Substances implicated in this relatively late developing vasospasm include vasoconstrictor factors released from and formed in response to the hemorrhaged blood, including inhibitors of nitric oxide, such as hemoglobin, and vasoconstrictors, such as endothelin-1, as well as bilirubin oxidation products (BOXes; Clark and Sharp, 2006; Crowley et al., 2008); it should be noted that the abbreviation, “BOXes,” was defined as “…the plural form of BOX A [4-methyl-5-oxo-3-vinyl-(1,5-dihydropyrrol-2-ylidene)acetamide] and BOX B [3-methyl-5-oxo-4-vinyl-(1,5-dihydropyrrol-2-ylidene)acetamide]” (Clark and Sharp, 2006), but has evolved to connote bilirubin oxidation products in general (e.g., Hou et al., 2011; Lakovic et al., 2014; Seidel et al., 2017). Indeed, seminal findings from the Clark laboratory (for reviews see, Clark and Sharp, 2006; Pyne-Geithman et al., 2013) provide key evidence related to the (1) presence of BOXes in the cerebral spinal fluid (CSF) of SAH patients and (2) ability of exogenous BOXes to elicit vasoconstriction. Thus, a pathway was proposed whereby BOXes are formed from the oxidation of heme which is eventually released from the hemorrhaged blood (Figure 1 as adapted from Clark and Sharp, 2006).

In fact, for the most part, these earlier studies (e.g., Kranc et al., 2000; Pyne-Geithman et al., 2005; Clark and Sharp, 2006) represent the sole findings with respect to this widely referenced hypothesis (e.g., Crowley et al., 2008; Siasios et al., 2013; Cheng et al., 2014; Wang et al., 2014, 2015; Foreman, 2016; Cseplo et al., 2017; Geraghty and Testai, 2017). Undoubtedly, contributing to the sparsity of follow-up studies with respect to the role of BOXes in SAH-induced spasm has been the difficulty in synthesizing sufficient amounts of chemically pure BOXes species implicated in vasospasm along with a sensitive assay for the measurement of endogenously formed BOXes species. However, in view of the recent total chemical synthesis of BOXes implicated in vasospasm, BOX A and BOX B (Klopfleisch et al., 2013; Seidel et al., 2014; **Figure 1**), along with sensitive assay methodology for their measurement (Seidel et al., 2015), it appears that the role of BOXes in SAH-induced spasm is at a juncture for significant further elucidation.

Thus, this perspective critically reviews the key findings supportive of a role for BOXes in SAH-induced vasospasm: the presence of BOXes in the CSF following SAH and the effects of exogenous BOXes on cerebral vascular contractility. Based on this critical evaluation, gaps remaining to be filled are identified and recent approaches that will address these gaps detailed. In this regard, for detailed descriptions of the mechanism whereby BOXes are formed following SAH and the associated pathobiologic effects other than vasoconstriction, including increased stress in neural and vascular tissue and cellular damage, as well as the role of BOXes in the pathobiology associated with another cerebral injury, intracerebral hemorrhage, the reader is referred to other reports and reviews (Clark and Sharp, 2006; Loftspring et al., 2010; Pyne-Geithman et al., 2013; Lakovic et al., 2014).

## Endogenous Boxes

The presence of vasoconstrictor factors in the CSF from SAH patients was suggested some four decades ago (Boullin et al., 1976). More than two decades later it was further suggested that these factors include BOXes (Kranc et al., 2000; Clark and Sharp, 2006). However, the sole report which attempted to identify the BOXes species potentially responsible for the vasoconstriction actually considered the compound identification as preliminary (Kranc et al., 2000). Specifically, assay of BOX A, BOX B, and MVM (4-methyl-3-vinylmaleimide; **Figure 1**) from CSF of SAH patients (extracted with chloroform followed by *in vacuo* evaporation of the chloroform, reconstitution in acetonitrile/water and subjected to reversed-phase HPLC) yielded peaks with similar retention times as BOX A, BOX B, and MVM (obtained from a reaction mixture containing bilirubin and hydrogen peroxide; data not shown; Kranc et al., 2000). Furthermore, doping the CSF with BOX A resulted in comigration with the corresponding peak (data not shown; Kranc et al., 2000). Indeed, it was concluded that “…the levels [of BOX A, BOX B, and MVM] so far observed have been too low to allow definitive identification.” and “Preliminary data suggest that these or similar compounds are present in the CSF of SAH patients” (Kranc et al., 2000). Additional limitations of the study (Kranc et al., 2000) included that the (1) source of CSF was not differentiated with respect to SAH patients symptomatic and asymptomatic for vasospasm and (2) quantitation of the amounts of (unconfirmed) BOX A and BOX B represented by the peaks could not be calculated due to the absence of their reported extinction coefficients (𝜀). Further, the MVM 𝜀 had not been established in solvent which eluted MVM from the reverse-phase HPLC column (acetonitrile:water; Kranc et al., 2000) and was restricted to earlier determinations in methanol (2,290, λ_max_ 319 nm and 2,300, λ_max_ 317 nm; Bonnett and McDonagh, 1969; Kurtin, 1978).

The limitations of this earlier report of elevated BOXes in the CSF of SAH patients (Kranc et al., 2000) were at least partially overcome in a subsequent study (Pyne-Geithman et al., 2005) in which (1) a comparison was performed between total BOXes levels in CSF extract from SAH patients asymptomatic and symptomatic for vasospasm, in which CSF only from symptomatic patients elicited constriction, and (2) total BOXes formation was estimated, with total BOXes CSF levels in asymptomatic patients at or below the lower limit of detection (0.007 μM was the lowest BOXes concentration reported) and in symptomatic patients total BOXes concentrations consistently somewhat greater than 1 μM (Pyne-Geithman et al., 2005; caveats associated with these concentration measurements discussed directly below). Moreover, the lower levels of BOXes in CSF from SAH patients asymptomatic for vasospasm was attributed to the comparatively lesser formation of bilirubin and weaker oxidizing environment, as based upon the lower CSF levels of malonyldialdehyde and peroxidized lipid (Pyne-Geithman et al., 2005, 2013). It should also be noted that bilirubin acts as a reducing agent (Gazzin et al., 2016). Thus, the greater oxidizing environment in CSF from SAH patients symptomatic for vasospasm occurred despite the greater CSF levels of bilirubin (Clark and Sharp, 2006).

In any case, due to the absence of sufficient purified compound, mole determinations were based upon a collective extinction coefficient (𝜀; 6,985 L/mol-cm^-1^) approximated using the absorption at 310 nm of a saline-reconstituted, chloroform extract of a hydrogen peroxide–bilirubin reaction mixture containing BOX A, BOX B, and MVM (Wurster et al., 2008). Caveats associated with adoption of absorption at 310 nm include that there was some variability in the amounts of these BOXes species formed and, in particular, varied formation of MVM (Wurster et al., 2008). This variability would influence the 𝜀 approximation because the λ_max_ for MVM is 317 or 319 nm, at least as determined in methanol (as noted above; Bonnett and McDonagh, 1969; Kurtin, 1978). Also, the λ_max_’s for BOX A and BOX B in saline are 300 and 310 nm, respectively (Kranc et al., 2000).

A second caveat associated with the approximated 𝜀 (Wurster et al., 2008) is that the determination was based upon an estimated 50% conversion of bilirubin to BOXes by the hydrogen peroxide oxidation of bilirubin, with the remaining 50% unreacted bilirubin and biliverdin as well as “other compounds” that do not absorb at 310 nm (Kranc et al., 2000; Pyne-Geithman et al., 2005; Wurster et al., 2008).

Finally, utilizing the approximated 𝜀 determined by total BOXes in a reconstituted chloroform extract of a hydrogen peroxide–bilirubin reaction mixture (Wurster et al., 2008) assumes that this product composition mimics the composition of endogenously formed BOXes. In this regard, the ratio of endogenous *Z*-BOX A:*Z-*BOX B is 1:1, as determined in human, rat, and mouse serum, and human bile and gallstones, as compared to 2:1 in the hydrogen peroxide–bilirubin reaction mixture (BOX A and BOX B can exist as *Z* and *E* regio-isomers, the *E* regio-isomers are not formed in the hydrogen peroxide–bilirubin reaction mixture; Seidel et al., 2015, 2017; Ritter et al., 2016; **Figure 1**). Also, it should be considered that relative amounts and species of endogenous BOXes formed may change with time post-SAH, the magnitude of SAH as well as factors that influence BOXes formation.

More recently, constriction of rat arteries in a cranial window preparation in response to CSF (unextracted) from two patients symptomatic for vasospasm was attributed to BOXes based on the (1) absorption of the CSF at 320 nm and (2) decreased constriction of arterioles of rat cortex, determined in a cranial window, following degradation (decreased amount of 320 nm absorption) of BOXes with exposure of the CSF to visible light (380–760 nm, 25 watts and 150 cm distance from sample; Sabanci et al., 2017). The attribution of BOXes degradation to the light-induced decrease in CSF-mediated constriction (Sabanci et al., 2017) was based upon earlier findings that ambient (t_1/2_ 10 h), sun-, and UV light caused decreased absorption of BOX A and BOX B in a reconstituted extract of a hydrogen peroxide–bilirubin reaction mixture (Kranc et al., 2000; Clark et al., 2008b; Wurster et al., 2008). Also, 30 min sunlight exposure isomerized *Z*-BOX A and *Z*-BOX B to their respective *E* regio-isomers and 8 h exposure caused considerable degradation (Seidel et al., 2015).

## Exogenous Boxes

Prior to 2014, by which time the complete chemical syntheses of both BOX A and BOX B had been reported (Klopfleisch et al., 2013; Seidel et al., 2014, respectively), and beginning with the initial report of BOXes (Kranc et al., 2000), the exogenous BOXes preparation used to determine biologic activity was derived from a lyophilized, 10% hydrogen peroxide–bilirubin reaction mixture (24 h incubation) reconstituted in physiologic saline. The reconstituted reaction mixture contained BOX A, BOX B, and MVM and, furthermore, at a 1.7:1.2:1 ratio (Wurster et al., 2008). This ratio was determined at the single wavelength of 320 nm (reversed-phase HPLC in acetonitrile/water eluate; Wurster et al., 2008) and, thus, reflects an approximation given the differing λ_max_’s/𝜀’s for these BOXes (Bonnett and McDonagh, 1969; Kurtin, 1978; Kranc et al., 2000; Klopfleisch et al., 2013; Harris et al., 2018) and the variability in the amounts of these BOXes species formed in the hydrogen peroxide–bilirubin reaction mixture (see above; Wurster et al., 2008). It should also be noted that the preparation presumably contained contaminants which did not absorb at 310 nm (Wurster et al., 2008).

The earliest report of BOXes-induced constriction utilized an *in vitro* preparation of the pig carotid artery and 1 mg/ml of BOXes (Kranc et al., 2000). The amount of actual BOXes was, in fact, significantly less than 1 mg/ml because the weight determination also included sodium chloride (originating from hydrochloric acid neutralization of the sodium hydroxide used to solubilize the bilirubin for oxidation by hydrogen peroxide; Kranc et al., 2000). An additional consideration is that the BOXes preparation consisted of a saline reconstituted, lyophilized hydrogen peroxide–bilirubin reaction mixture (Kranc et al., 2000). Thus, it was assumed that the hydrogen peroxide, which can elicit constriction, was removed by lyophylization (Kranc et al., 2000). As a control for possible contaminant effects, the extract was exposed to sunlight (90 min) to degrade BOX A, BOX B, and MVM (as described above in “Endogenous BOXes; Kranc et al., 2000; Clark et al., 2008b; Wurster et al., 2008; Seidel et al., 2015). Indeed, sunlight decreased the biological activity of the reconstituted BOXes mixture (Kranc et al., 2000). While the decreased biologic activity of the reconstituted BOXes mixture due to sunlight also appears consistent with the activity of the BOXes preparation not involving possible remaining peroxides, on the other hand it should be considered that sunlight/UV light degrades hydrogen peroxide.

In apparent contrast to this earlier finding that reconstituted BOXes mixture constricted pig carotid artery (Kranc et al., 2000; note that CSF extract also constricted pig carotid artery; Pyne et al., 2000, 2001; Pyne-Geithman et al., 2008), a subsequent study with this vessel *in vitro* failed to demonstrate constriction in response to a BOXes mixture approximated at 20 μM (calculated with an estimated 𝜀 of 6,985 L/mol-cm^-1^ derived as described above in “Endogenous BOXes”; Pyne-Geithman et al., 2008). While the reason underlying these apparently contrasting findings is not clear, a possible explanation is that the BOXes preparation utilized in the subsequent study was extracted with chloroform prior to use (Pyne-Geithman et al., 2008). Specifically, the hydrogen peroxide–bilirubin reaction mixture that had been neutralized with hydrochloric acid and then lyophilized was extracted with chloroform and then dried under nitrogen prior to saline reconstitution (Pyne-Geithman et al., 2008). Chloroform extraction would have substantially reduced the amount of sodium chloride in the BOXes preparation (Pyne-Geithman et al., 2008) as well as possible hydrogen peroxide remaining following lyophilization.

Despite the inability of the reconstituted BOXes mixture to constrict the pig carotid artery in this subsequent study, the mixture elicited a non-selective increase in contractile sensitivity of this vessel (Clark and Sharp, 2006; Pyne-Geithman et al., 2008). Specifically, the BOXes mixture enhanced contractions to phenylephrine, histamine, and KCl (Clark and Sharp, 2006; Pyne-Geithman et al., 2008). The mechanism underlying the enhanced constriction may have been due to the observed BOXes-mediated increased activity of protein kinase C α and δ and Rho A, while purified protein phosphatase 1 activity remained unaltered (Pyne-Geithman et al., 2008). It should also be noted that CSF extract of SAH patients symptomatic for vasospasm activated protein kinase C α and δ and Rho A (Pyne-Geithman et al., 2008). The mechanism whereby the reconstituted BOXes mixture, as well as the CSF extract of SAH patients symptomatic for vasospasm, activate these two signaling pathways remains to be determined.

With respect to BOXes constriction of cerebral vessels, in a cranial window placed over the rat parietal cortex, differing effects of the supradural application (single dose) of a reconstituted BOXes mixture on vascular tone were observed depending on the dilution of the BOXes containing solution (Clark et al., 2002). While the least dilution (greatest concentration) of the BOXes mixture caused dilation and the greatest dilutions lacked both constrictor and dilator efficacy, intermediate dilutions caused constriction (Clark et al., 2002; vehicle controls not reported). The constriction was relatively slow to develop, i.e., 40 min, and was still present at 24 h (Clark et al., 2002), consistent with the prolonged vasospasm observed with SAH (Crowley et al., 2008; Siasios et al., 2013; Flynn et al., 2017; Geraghty and Testai, 2017).

Possibly related to the mechanism underlying the reconstituted BOXes mixture-induced constriction (Kranc et al., 2000; Clark et al., 2002), a BOXes mixture rapidly and concentration-dependently (1–100 μM, estimated with the approximated 𝜀 derived in Wurster et al., 2008) diminished the open probability of Slo1 K^+^ channels as determined by patch clamp of myocytes isolated from the mouse basilar artery and branches (Hou et al., 2011). The BOXes mixture utilized by Hou et al. (2011) was reported as containing BOX A:BOX B:MVM at a 2:2:1 ratio, a ratio presumably based upon relative peak absorption at 310 nm (Wurster et al., 2008). Sunlight prevented the effect of the reconstituted BOXes mixture on Slo1 K^+^ channels, consistent with the action of BOXes rather than a contaminant (Hou et al., 2011). The BOXes mixture was effective when added to either the extracellular or cytoplasmic side, although the response was more rapid on the latter (Hou et al., 2011). Additionally, the effect of BOXes was slow to reverse (Hou et al., 2011). These findings of Hou et al. (2011) are consistent with the ability of BOXes to traverse the membrane to act at a cytoplasmic site of Slo1 K^+^ channels and, further, the sustained constriction following supradural application of BOXes (Clark et al., 2002), the resistance to reversal of the constriction of cerebral vessels in brain slices (see below; Joerk et al., 2014), and the prolonged vasospasm following SAH (Crowley et al., 2008; Siasios et al., 2013; Foreman, 2016; Flynn et al., 2017; Geraghty and Testai, 2017). In addition to a direct inhibitory effect of BOXes on Slo1 K^+^ channels (Hou et al., 2011), BOXes activation of protein kinase C (Pyne-Geithman et al., 2008) may represent an additional signaling pathway for Slo1 K^+^ channel inhibition. In support of this suggestion are demonstrations of protein kinase C inhibition of Slo1 K^+^ channel inhibition in vascular smooth muscle, although protein kinase C activation of Slo1 K^+^ channel has also been reported (Kim and Park, 2008 and references therein).

More recently, in mouse brain slices, 5 μM *Z*-BOX A (purified by reversed-phase HPLC) constricted intracerebral arterioles (pre-constricted with a nitric oxide synthase inhibitor) of the visual cortex (Joerk et al., 2014). In comparison, 5 μM *Z*-BOX B (purified by reversed-phase HPLC) was much less efficacious and elicited a slower time course for constriction (although the possibility was raised that a longer period of *Z*-BOX B exposure could potentially elicit a greater magnitude of constriction; Joerk et al., 2014). The *Z*-BOX A-induced plateau constriction required approximately 1 h to develop and was maintained for at least 30 min following washout (Joerk et al., 2014), findings qualitatively similar to the relatively slow onset of constriction and maintained constriction of superficial vessels of the rat parietal cortex following supradural application of a single dose of BOXes mixture (Clark et al., 2002). Also, consistent with the ability of the BOXes mixture to diminish the open probability of Slo1 K^+^ channels (Hou et al., 2011), Slo1 K^+^ channel knockout prevented *Z*-BOX A-induced arteriolar constriction (Joerk et al., 2014). The ability of *Z*-BOX A to elicit constriction appears to be vessel specific in that *Z*-BOX A failed to increase pressure in an isolated liver perfusion preparation of the rat (Seidel et al., 2017).

In terms of possible additional targets for BOXes, in hepatocytes *Z*-BOX A and *Z*-BOX B were cytotoxic, initiated cytoskeletal remodeling, influenced the glutathione redox state, modulated *Rev-erb*α/β activity (regulation of metabolism of endogenous and exogenous compounds), and decreased CYP7A1 in its capacity as the rate limiting enzyme in the synthesis of bile acids (Seidel et al., 2017). However, whether any of these findings in hepatocytes are transferrable to the pathobiologies associated with BOXes in SAH remains for further investigation.

## Conclusion, Speculations, and Future Directions

Findings support the presence of BOXes in the CSF of SAH patients and, moreover, are associated with CSF from patients symptomatic for vasospasm (Pyne-Geithman et al., 2005; Sabanci et al., 2017). Further, it appears that the concentration of BOXes in the CSF of these patients is sufficient to contribute to the vasospasm (Pyne-Geithman et al., 2005; Sabanci et al., 2017). Clearly, the concentrations of the different BOXes species and the time courses for their formation remain to be established. Along these lines, it was estimated that total BOXes formation in patients symptomatic for vasospasm was approximately 1 μM (Pyne-Geithman et al., 2005), a concentration in a similar range as the concentration of *Z*-BOX A that constricted intracerebral arterioles in brain slices of the mouse visual cortex (Joerk et al., 2014). It should also be considered that additional BOXes species are vasoactive as well as enhance constriction to other agonists which are associated with SAH-induced vasospasm (Clark and Sharp, 2006; Pyne-Geithman et al., 2008), including endothelin-1 (Crowley et al., 2008).

Interestingly, there was a log-log (linear), 1000:1 correlation between bilirubin and *Z*-BOX A plus *Z*-BOX B plasma levels, with the greatest bilirubin concentration assayed approximately 0.6 μM, as determined in healthy humans and patients with cholestatic liver failure and hereditary deficiency of bilirubin glucuronidation in rats (Seidel et al., 2017). Based upon the amount of bilirubin in the CSF of SAH patients symptomatic for vasospasm of approximately 30 μM (Pyne-Geithman et al., 2005) and extrapolation of the correlation between bilirubin and *Z*-BOX A plus *Z*-BOX B plasma levels (Seidel et al., 2017) it would be predicted that 30 nM *Z*-BOX A plus *Z*-BOX B is present in the CSF of these patients. Also, with accounting for the lack of vasoconstrictor activity of *Z*-BOX B, the predicted concentration of *Z*-BOX A in CSF from SAH patients symptomatic for vasospasm would be approximately two orders of magnitude less than the concentration which constricted intracerebral arterioles in brain slices of the mouse visual cortex (5 μM; Joerk et al., 2014). A likely explanation for this difference in predicted concentration of *Z*-BOX A and the concentration which elicited arteriole constriction in brain slices (Joerk et al., 2014) is that the cerebral oxidizing environment following SAH in these patients (Pyne-Geithman et al., 2005, 2013) is considerably greater than the oxidizing environment in the liver under the conditions which demonstrate a 1000:1 correlation between bilirubin and *Z*-BOX A plus *Z*-BOX B (Seidel et al., 2017).

In terms of the presence of BOXes species in biologic compartments in addition to CSF following SAH, it would be anticipated that levels of BOXes in the CSF reflect those in the blood clot and, moreover, at least at relatively earlier times post-blood clot formation are present in the clot at a comparatively higher concentration than the CSF. Also, the lipid solubility of BOXes, as evidenced by extraction in chloroform (Kranc et al., 2000; Clark and Sharp, 2006; Wurster et al., 2008; Ritter et al., 2016; Harris et al., 2018), as well as the ability of supradural application of BOXes to access superficial cortical vessels (Clark et al., 2002), i.e., a phenomenon which requires traversal of BOXes through the dura and arachnoid membranes (Bernards and Hill, 1992), and the octanol/water partition coefficients (log P_OW_) of *Z*-BOX A and *Z*-BOX B of 0.92 and 1.11, respectively (solubility in water of 103.6 and 76.4 mg/ml, respectively; Seidel et al., 2017), suggest that BOXes derived from the clot can accumulate in the tissues. Of some possible relevance to this suggestion are findings with intracellular hemorrhage in a pig model, in which the perihematomal tissue demonstrated elevated levels of BOXes (Clark et al., 2008a). On the other hand, recovery of *Z*-BOX A and *Z*-BOX B from plasma was 90 and 92%, respectively, utilizing a polar solvent (acetonitrile:water, 2:98 v/v; Seidel et al., 2015).

It should also be anticipated that the formation of different BOXes species (with varying constrictor potencies and efficacies), as well as their levels of formation, depends upon the magnitude/site of injury. Thus, the species and levels formed would depend upon the oxidative environment. In this regard, propentdyopents, precursor isomers of *Z*-BOX A and *Z*-BOX B, were formed as a result of bilirubin oxidation with a lower concentration of hydrogen peroxide (1% as compared to 10%) and, moreover, were detected in human gallstones and bile of patients undergoing cholystectomy (Ritter et al., 2016). Furthermore, propentdyopents levels were significantly greater than *Z*-BOX A and *Z*-BOX B levels in the bile of these patients (Ritter et al., 2016). More recently, the formation of “*Z*-BOX C”, a propentdyopent product, was detected in bile from patients with cholecystectomy (Ritter et al., 2018). Thus, it would be of interest whether the propentdyopents and *Z*-BOX C are efficacious vasoconstrictors, in addition to other possible effects. Additionally, oxidation of bilirubin with storage (4 and 37°C for 2 and 7 days) resulted in the formation of BOXes species other than BOX A, BOX B, MVM and propentdyopents, and which remain unidentified (Lakovic et al., 2014; Ritter et al., 2016). Intracerebral infusion of this BOXes preparation combined with bilirubin elicited greater pathobiologic effects than bilirubin alone (Lakovic et al., 2014). Thus, it is also possible that one or more of the BOXes formed upon storage possess vasoconstrictor activity. Further, as the oxidative environment in SAH is influenced by the amount of hemorrhage, it would be predicted that the formation of different BOXes species depends upon the amount of blood clot formation.

It may also be considered that the vasoconstriction (as well as other effects) elicited by crude extract of the bilirubin-hydrogen peroxide reaction mixture (Clark and Sharp, 2006; Pyne-Geithman et al., 2013), could have been at least partly *Z*-BOX A-mediated based on the (1) selective cerebral vasoconstrictor effect of *Z*-BOX A and (2) preferential formation of *Z*-BOX A as compared to *Z*-BOX B (2:1) by hydrogen peroxide (10%) oxidation of bilirubin (Seidel et al., 2017). Further, the estimated concentrations of BOXes mixture which constricted cerebral arterioles in a cranial window (20 μM; Clark et al., 2002) and decreased Slo1 K^+^ channel opening (1–100 μM; Hou et al., 2011), are within the range of the *Z*-BOX A concentration that constricted arterioles in brain slices (5 μM; Joerk et al., 2014).

In terms of the mechanism underlying BOXes-induced vasoconstriction, the (1) relatively slowly developing contractile response and maintained constriction elicited by *Z*-BOX A (Joerk et al., 2014) and BOXes mixture (Clark et al., 2002) and (2) ability of BOXes mixture to traverse the extracellular membrane to a cytoplasmically located portion of the Slo1 K^+^ channel and also slow reversal of the BOXes effect on this channel (Hou et al., 2011), may suggest that the efficacious BOXes species, as the result of its lipid solubility [e.g., octanol–water partition coefficients (log P_OW_) for *Z*-BOX A and *Z*-BOX B of 0.92 and 1.11, respectively; Kranc et al., 2000; Wurster et al., 2008; Seidel et al., 2017], becomes inserted into the plasma membrane whereupon it acts at a specific site. Further, the maintained constriction could result from the continued inhibition (e.g., the diminished probability of Slo1 K^+^ channel opening; Hou et al., 2011; Joerk et al., 2014) of this signaling mechanism due to the continued presence of the BOXes species in the membrane. As an additional speculation, the membranal insertion of *Z*-BOX A would also prevent its further oxidative-induced degradation, thereby also contributing to a prolonged effect.

With respect to the concentration-dependent selectivity of BOXes, the ability of >100 μM of both *Z*-BOX A and *Z*-BOX B to increase hepatocyte toxicity suggest that higher concentrations of *Z*-BOX A lack selectivity (Seidel et al., 2017). On the other hand, >100 μM concentrations of *Z*-BOX A, but not *Z*-BOX B, caused morphological changes in the HepG2 cells (Seidel et al., 2017). These results, along with the finding that *Z*-BOX A constricts cerebral vessels (Joerk et al., 2014), but not hepatic arteries (Seidel et al., 2017), suggest that *Z*-BOX A and *Z*-BOX B action depends upon different targets with differing affinities. Along these lines, whether the inability of *Z*-BOX A to increase liver perfusion pressure (Seidel et al., 2017) is due to a relative minimal number of Slo1 K^+^ channels in hepatic resistance vessels also remains for consideration.

Laying the groundwork for filling these gaps are recent notable advances in this field, including total synthesis of the *Z*-BOX regio-isomers (Klopfleisch et al., 2013; Seidel et al., 2014). Amongst other advances, this synthesis would assist in the isotopic labeling of significant amounts of these regio-isomers for binding studies. Also, the recently developed sensitive assays for the *Z*-BOX regio-isomers and the novel precursors to *Z*-BOX A and *Z*-BOX B, the propentdyopents, will provide measurements of endogenous formation and concentrations of these BOXes in relevant biologic compartments (Seidel et al., 2015; Ritter et al., 2016). Filling these gaps will assist in determinations of the mechanism of BOXes-induced vasoconstriction and, thus, the eventual development of pharmacologic agents which selectively antagonize the constriction as well as other pathobiologic effects.

## Author Contributions

The author confirms being the sole contributor of this work and approved it for publication.

## Conflict of Interest Statement

The author declares that the research was conducted in the absence of any commercial or financial relationships that could be construed as a potential conflict of interest.
